# Real-world treatment patterns and outcomes for patients with non-metastatic non-small cell lung cancer: retrospective analyses in Canada, England, and Germany

**DOI:** 10.1186/s12890-025-03715-9

**Published:** 2025-05-27

**Authors:** Alastair Greystoke, Melinda J. Daumont, Caroline Rault, Hannah Baltus, Philip Q. Ding, Gabrielle Emanuel, Stefano Lucherini, Lien Vo, Valeria M. Saglimbene, Eleanor Ralphs, Cátia Leal, Minouk J. Schoemaker, Alexander Katalinic, Annika Waldmann, Winson Y. Cheung

**Affiliations:** 1 Newcastle Hospital and Newcastle University, Newcastle Upon Tyne, UK; 2 Sir Bobby Robson Clinical Trials Unit, Freeman Hospital, Newcastle Upon Tyne, UK; 3 Worldwide Health Economics & Outcomes Research, Bristol Myers Squibb, Braine-L’Alleud, Belgium; 4 Data Gnosis, Rennes, France; 5 Institute for Social Medicine and Epidemiology, University of Lübeck, Lübeck, Germany; 6 Department of Oncology, University of Calgary, Oncology Outcomes, Calgary, AB Canada; 7 Real World Data Analytics Markets, Bristol Myers Squibb, Uxbridge, UK; 8 Worldwide Health Economics & Outcomes Research, Bristol Myers Squibb, Uxbridge, UK; 9 Worldwide Health Economics & Outcomes Research, Bristol Myers Squibb, Princeton, NJ USA; 10 Real World Solutions, IQVIA Ltd, Milan, Italy; 11 Real World Solutions, IQVIA Ltd, London, UK; 12 Real World Solutions, IQVIA Ltd, Lisbon, Portugal; 13 Real World Solutions, IQVIA Ltd, Amsterdam, Netherlands

**Keywords:** I-O Optimise, Real-world evidence, Database, Registries, Survival, Treatment patterns

## Abstract

**Background:**

Recent therapeutic advancements for non-metastatic non-small cell lung cancer (NSCLC) have increased the need for real-world baselines against which future changes in patient management and clinical outcomes can be compared.

**Methods:**

Data on patient characteristics, initial treatment, and overall survival (OS) were derived from adult patients diagnosed with stage I-IIIC NSCLC (2010–2020) in a regional Canadian database (Oncology Outcomes [O2]), an English national registry (Cancer Analysis System [CAS]), and four regional German registries (VONKOdb) and retrospectively analyzed separately using analogous methodology.

**Results:**

Data from 85,433 patients were analyzed. Stage at diagnosis varied, with proportions with stage I NSCLC ranging from 30.9% (VONKOdb) to 44.2% (O2) and with stage III disease from 36.9% (O2) to 48.5% (VONKOdb). Across the data sources, proportions receiving surgery ± other treatments were similar for stages I and II, but decreased through stages IIIA, IIIB, and IIIC (range, 24.7–42.7%, 4.6–21.8%, and 0.9–7.5%, respectively). Overall, 70.3–85.2% of patients received active treatment for NSCLC, with a trend toward lower proportions among those with stage III disease. Reached median OS tended to be longest in patients with resected stage I/II NSCLC (range, 28.8–128.0 months) and shortest in patients with stage IIIB/IIIC disease treated with systemic anticancer therapy (SACT) alone, radiotherapy alone, or SACT + palliative radiotherapy (range, 4.8–21.2 months).

**Conclusions:**

These data provide insights into treatment pathways and survival outcomes before the widespread use of immunotherapy-based and targeted therapies and will serve as an important baseline for future evaluations of emerging treatments for patients with non-metastatic NSCLC.

**Supplementary Information:**

The online version contains supplementary material available at 10.1186/s12890-025-03715-9.

## Introduction

Based on the latest Global Cancer Observatory GLOBOCAN estimates (2022), lung cancer accounts for around 10% of the approximately 7 million emergent cancer cases in Europe and North America and is the leading cause of cancer death in these regions and worldwide [1–3]. Non-small cell lung cancer (NSCLC) is the most common type of lung cancer, and approximately 40−50% of patients with NSCLC are diagnosed at non-metastatic stages of the disease (American Joint Committee on Cancer [AJCC] and Union for International Cancer Control [UICC] tumor, node, metastasis [TNM] classification system stages I to III) [4–8], with approximately 20–30% diagnosed with early-stage NSCLC (stages I or II) and approximately 20% diagnosed with locally advanced disease (stage III) [4, 6, 7].

Historically, European and North American treatment guidelines for non-metastatic NSCLC have recommended surgery with curative intent for eligible patients with resectable early-stage or locally advanced tumors, with neoadjuvant and/or adjuvant chemotherapy advised in certain patient populations [9–12]. Among patients with unresectable tumors or among those who refuse surgery or are inoperable because of their general condition, radiotherapy (RT) with curative intent has been recommended for early-stage NSCLC and concurrent or sequential chemoradiotherapy (CRT) for locally advanced disease [9–11]. For patients with locally advanced NSCLC who are considered unfit for treatment with curative intent, options have included palliative RT, palliative chemotherapy, or best supportive care [13–16], although, in certain countries/regions, select patients with stage III disease might also have been able to receive immunotherapy-based and targeted therapies approved in the advanced/metastatic NSCLC setting.

More recently, the therapeutic landscape for non-metastatic NSCLC has expanded with the emergence of new treatment options and modalities. Although specific approvals vary between Europe and North America, select patient populations with resectable tumors now have access to novel perioperative treatment regimens such as a combination of nivolumab (a programmed death 1 [PD-1] inhibitor) and platinum-doublet chemotherapy in the neoadjuvant setting, atezolizumab or pembrolizumab (PD-L1 and PD-1 inhibitors, respectively) monotherapy in the adjuvant setting, and pembrolizumab in the perioperative setting; osimertinib (an epidermal growth factor receptor [EGFR] tyrosine kinase inhibitor) is also an adjuvant option for *EGFR*-mutated NSCLC [17–21]. Likewise, patients with unresectable locally advanced NSCLC whose disease has not progressed after concurrent platinum-based CRT can receive consolidation therapy with durvalumab (a PD-L1 inhibitor) or osimertinib (if carrying *EGFR*-mutated NSCLC) according to specific regional guidance [17, 21–24].

These fast-paced changes to the treatment options for non-metastatic NSCLC create a need to evaluate novel therapies and strategies in real-life settings to support findings of the respective clinical trials and ultimately guide real-world treatment decision-making. An important part of such an evaluation is the establishment of a real-world baseline against which future changes in patient management and clinical outcomes can be compared, allowing accurate measurement of the effect of the introduction of new treatment approaches.

I-O Optimise is an ongoing, multi-country, collaborative research initiative that leverages existing real-world cancer databases to provide insights into the management of thoracic malignancies in clinical practice [25]. To date, data on patients with NSCLC, small cell lung cancer, and malignant pleural mesothelioma from various data sources in Europe and Canada have been analyzed as part of the I-O Optimise initiative [5–7, 26–35]. Using data collected in Canada, England, and Germany as part of the I-O Optimise initiative, the current retrospective analyses aimed to describe real-world patient characteristics, initial treatment patterns, and overall survival (OS) outcomes among patients diagnosed with non-metastatic NSCLC from 2010 through 2020, thus establishing a comprehensive real-world baseline for future evaluation of the impact of newer treatment options on patient outcomes.

## Patients and methods

### Data sources

Data were derived from studies performed at three registry-based data sources: (1) the Oncology Outcomes (O2) database, a regional registry capturing cancer-related data for the province of Alberta in Canada, representing approximately 4.5 million residents [31]; (2) the Cancer Analysis System (CAS) registry, a national registry capturing data on all cancer cases where medical care is provided by the National Health Service (NHS) among the population of England (approximately 55 million people during the inclusion period of this study) [29, 36, 37]; and (3) the Oncological Health Care Research Database (VONKOdb), a cancer-related database comprising pooled data from four regional population-based clinical cancer registries for the federal states of Hamburg, Baden-Württemberg, North Rhine-Westphalia, and Schleswig–Holstein in Germany, representing approximately 33 million residents and approximately 40% of the German population. Further details on these data sources are provided in the Supplementary Methods.

### Patients

Eligible patients in all three data sources were adults (≥ 18 years) with an incident diagnosis of lung cancer between 2010 and 2020 per specified International Classification of Diseases and Related Health Problems, 10 th Revision (ICD-10) diagnostic codes (Supplementary Table 1); a specified International Classification of Diseases for Oncology, 2nd edition (ICD-O-2) or 3rd edition (ICD-O-3) morphology code corresponding to the ‘adenocarcinoma’, ‘squamous cell carcinoma’, ‘NSCLC not otherwise specified’ (NOS), ‘large cell carcinoma’, or ‘other miscellaneous NSCLC’ histological groups; and recorded stage I-III disease (including relevant sub-staging for stages IA, IB, IIA, IIB, IIIA, IIIB, or IIIC) at the date of NSCLC diagnosis. Disease staging was based on the 7 th or 8 th edition of the TNM staging classification system per the AJCC/UICC, depending on the year of diagnosis [38–41]; stage IIIC was only introduced in the 8 th edition, which was officially utilized in the data sources from 2017/2018 onward. There were no restrictions on the source data used for TNM staging (clinical vs. post-surgical/pathological staging). Per the study protocols for all data sources, patients would be excluded if information on age and/or sex at diagnosis was missing. Specific exclusion criteria for CAS were a missing NHS number, a primary malignancy in the 5 years before lung cancer diagnosis date, treatment with any systemic anticancer therapy (SACT) within the period from 2 years before until 30 days before lung cancer diagnosis, or receipt of any drug in the United Kingdom Cancer Drug Fund at the time of data analysis. Specific exclusion criteria for VONKOdb were 0 days of follow-up, which encompasses true death certificate–only cases (i.e., patients who were only identified by the registry via their death certificate); a follow-up endpoint recorded before the recorded date of lung cancer diagnosis; or a missing/unknown vital status date.

### Study design and analyses

**Fig. 1:**
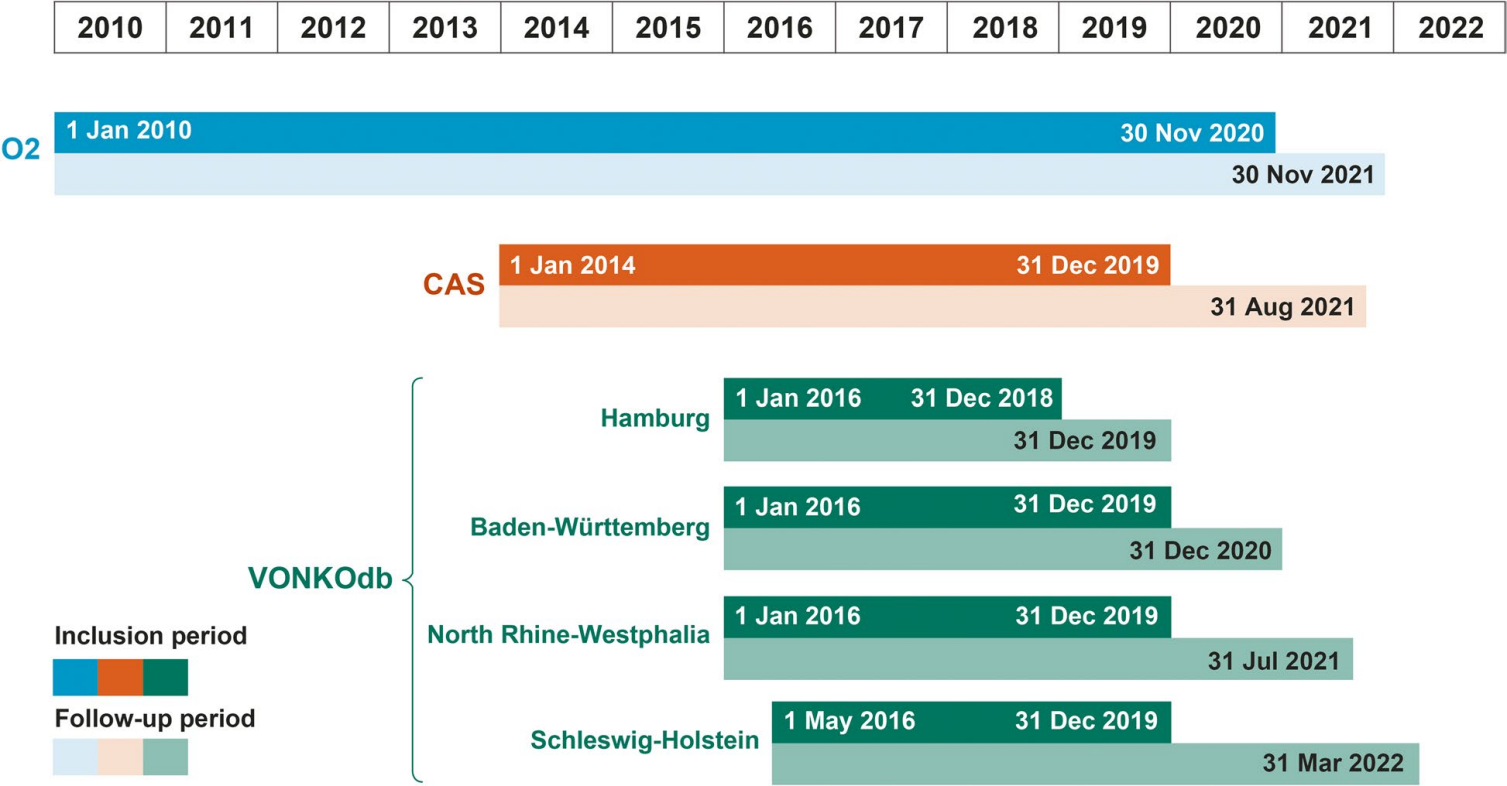
Study inclusion and follow-up periods. CAS, Cancer Analysis System; O2, Oncology Outcomes; VONKOdb, Oncological Health Care Research Database

For each data source–specific study, patients were included at date of diagnosis and followed until the earliest of death, known exit from data source (e.g., lost to follow-up), or end of study (end date of follow-up). Inclusion and follow-up periods are detailed in Fig. 1. Analyses were stratified by NSCLC stage at diagnosis (I, II, IIIA, IIIB, IIIC) and initial treatment ‘type’ or ‘category’ where applicable. Initial treatment ‘category’ was derived algorithmically for each data source using a similar algorithmic approach specifically designed to identify initial treatment received that was developed as part of the I-O Optimise initiative (Supplementary Fig. 1 and Supplementary Table 2). Initial treatment ‘type’ represents a classification based on assigned treatment category and included ‘resected’ (receiving surgery with or without other treatments such as adjuvant, neoadjuvant, or perioperative SACT and/or RT), ‘unresected’ (receiving only non-surgical treatments), ‘untreated’ (no receipt of surgery, RT, and/or SACT, but receipt of best supportive care), or ‘unclassified’ (receiving treatments that did not fit into any of the algorithmically derived treatment definitions categories [e.g., due to incomplete information or treatments appearing in a sequence or with time intervals that were not captured in the algorithmic rules]).

Data were analyzed separately for the three data sources using analogous methodology, with no pooling of data. Patient demographic and clinical characteristics were collected at inclusion date (diagnosis) and summarized using descriptive statistics. OS was estimated from the specified index date (diagnosis, start of treatment, or specific landmark time points in conditional survival analyses) using Kaplan–Meier methods. Conditional survival analyses assessed the probability of a patient surviving for a further 2 years after reaching sequential survival landmarks of 1, 3, or 5 years after diagnosis. In all OS analyses, patients who were alive were censored at end of the study periods or known exit from the data source. Data masking was conducted per data source–specific confidentiality requirements. Primary data masking was performed if patient counts for individual categories were between 1 and 9 for O2, between 1 and 5 for CAS, or between 1 and 4 for VONKOdb. For all data sources, additional secondary data masking was conducted where necessary to prevent unmasking of those categories undergoing primary data masking.

## Results

### Patient characteristics

**Fig. 2:**
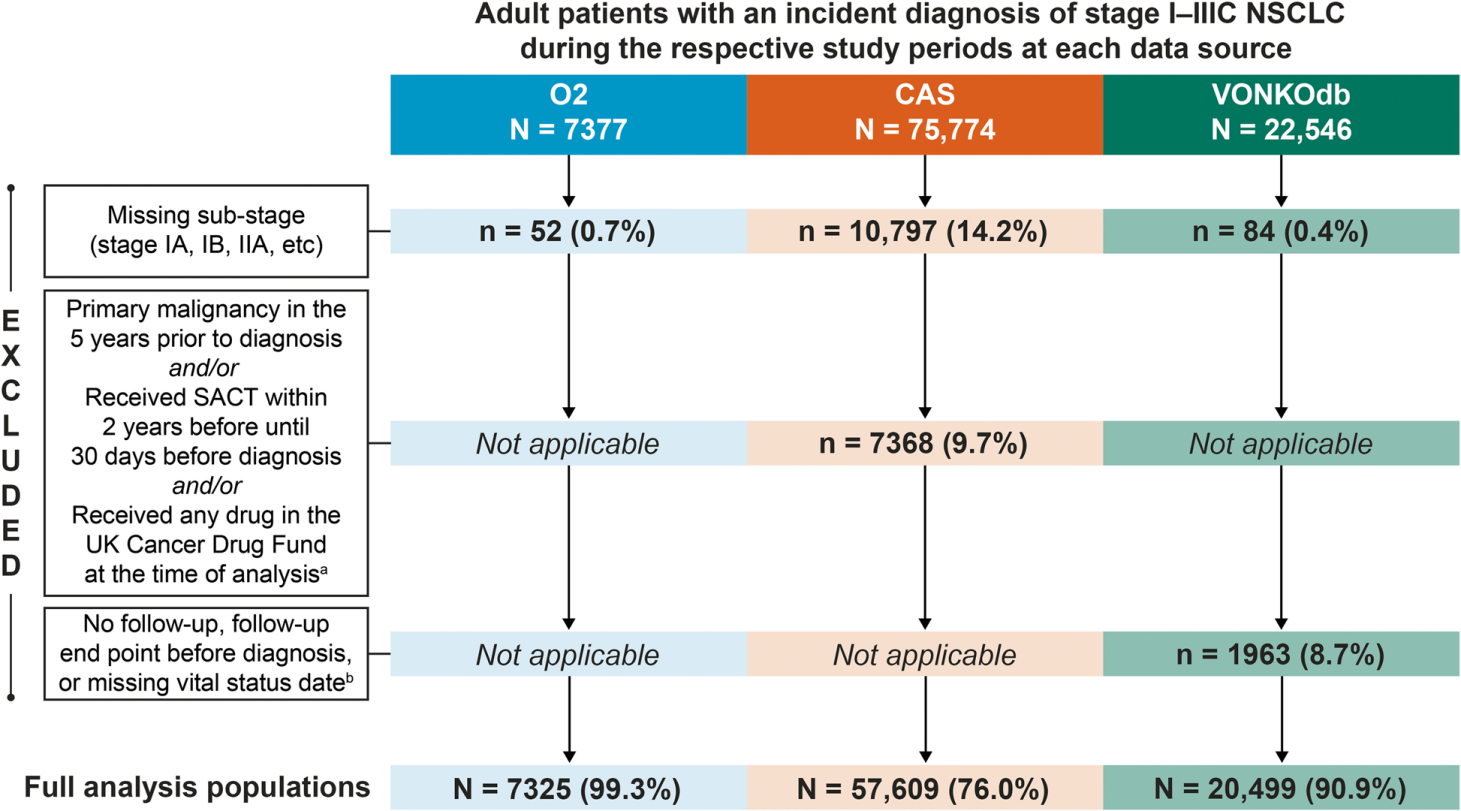
Patient flow chart. ^a^ Exclusion criteria specific to the CAS data source. ^b^Exclusion criteria specific to the VONKOdb data source. CAS, Cancer Analysis System; NSCLC, non-small cell lung cancer; O2, Oncology Outcomes; SACT, systemic anticancer therapy; VONKOdb, Oncological Health Care Research Database

In total, 85,433 eligible patients diagnosed with non-metastatic NSCLC were included in the studies conducted for O2 (*N* = 7325), CAS (*N* = 57,609), and VONKOdb (*N* = 20,499) (Fig. 2). Key patient and clinical characteristics are shown by disease stage at diagnosis in Table 1. Across the data sources, there was variability in disease stage at diagnosis, with the proportion of patients with stage I NSCLC highest for O2 (44.2%) and lowest for VONKOdb (30.9%) and the proportion with stage III disease highest for VONKOdb (48.5%) and lowest for O2 (36.9%). There were also differences between data sources in terms of distribution by sex (smaller proportions of female patients for VONKOdb) at all disease stages, and age (younger age for VONKOdb) and histology (smaller proportions with squamous histology and larger proportions with NOS for O2) at stages I through IIIB (Table 1). It was also noteworthy that within each data source there were trends toward younger age, decreasing proportions of female patients, and decreasing proportions of patients with non-squamous histology (parallel with increasing proportions with squamous histology) as disease stage increased (Table 1). Since stage III NSCLC represents a heterogenous entity, additional data on tumor and node stage distribution are shown in Supplementary Table 3. It is noteworthy that proportions of patients with stage IIIA-N2 disease were higher for O2 (64.7%) and CAS (55.9%) than for VONKOdb (45.7%) and proportions of patients with stage IIIB-N3 disease were also higher for O2 (47.9%) and CAS (44.4%) than for VONKOdb (28.9%) (Supplementary Table 3).

**Table 1:** Key characteristics of eligible patients with stage I-IIIC NSCLC from O2, CAS, and VONKOdb^a^

	**O2 (Canada) *N* = 7325**	**CAS (England) *N* = 57,609**	**VONKOdb (Germany) *N* = 20,499**
**Stage I,** *n *(%)^**b**^	***n* = 3236 (44.2)**	***n* = 20,994 (36.4)**	***n* = 6333 (30.9)**
Age, years			
Median (IQR)	71 (64–77)	72 (66–78)	69 (62–76)
Age ≥ 65 years, n (%)	2383 (73.6)	16,574 (78.9)	4303 (67.9)
Female, *n *(%)	1919 (59.3)	11,474 (54.7)	2680 (42.3)
Histology, *n *(%)			
Non-squamous	1973 (61.0)	12,939 (61.6)	3717 (58.7)
Adenocarcinoma	1957 (60.5)	12,647 (60.2)	3686 (58.2)
Squamous	690 (21.3)	5769 (27.5)	1903 (30.0)
NOS	486 (15.0)	1853 (8.8)	476 (7.5)
Other	87 (2.7)	433 (2.1)	237 (3.7)
Sub-stage, *n *(%)			
IA	1918 (59.3)	11,915 (56.8)	4282 (67.6)
IB	1318 (40.7)	9079 (43.2)	2051 (32.4)
**Stage II, ***n *(%)^**b**^	***n* = 1389 (19.0)**	***n* = 11,127 (19.3)**	***n* = 4230 (20.6)**
Age, years			
Median (IQR)	71 (64–78)	72 (66–79)	69 (62–76)
Age ≥ 65 years, *n *(%)	1016 (73.1)	8777 (78.9)	2857 (67.5)
Female, *n *(%)	701 (50.5)	4935 (44.4)	1509 (35.7)
Histology, *n *(%)			
Non-squamous	671 (48.3)	5125 (46.1)	1950 (46.1)
Adenocarcinoma	656 (47.2)	4954 (44.5)	1931 (45.7)
Squamous	496 (35.7)	4832 (43.4)	1845 (43.6)
NOS	165 (11.9)	811 (7.3)	254 (6.0)
Other	57 (4.1)	359 (3.2)	181 (4.3)
Sub-stage, n (%)			
IIA	569 (41.0)	4751 (42.7)	1404 (33.2)
IIB	820 (59.0)	6376 (57.3)	2826 (66.8)
**Stage IIIA, ***n *(%)^**b**^	***n* = 1656 (22.6)**	***n* = 14,295 (24.8)**	***n* = 5083 (24.8)**
Age, years			
Median (IQR)	71 (63–78)	72 (65–78)	68 (61–75)
Age ≥ 65 years, n (%)	1186 (71.6)	10,984 (76.8)	3254 (64.0)
Female, *n *(%)	795 (48.0)	6294 (44.0)	1767 (34.8)
Histology, *n *(%)			
Non-squamous	730 (44.1)	6110 (42.7)	2308 (45.4)
Adenocarcinoma	715 (43.2)	5943 (41.6)	2277 (44.8)
Squamous	613 (37.0)	6714 (47.0)	2163 (42.6)
NOS	257 (15.5)	1154 (8.1)	390 (7.7)
Other	56 (3.4)	317 (2.2)	222 (4.4)
**Stage IIIB, ***n *(%)^**b**^	***n* = 958 (13.1)**	***n* = 10,026 (17.4)**	***n* = 3780 (18.4)**
Age, years			
Median (IQR)	69 (63–76)	71 (64–77)	68 (61–75)
Age ≥ 65 years, *n *(%)	653 (68.2)	7395 (73.8)	2302 (60.9)
Female, *n *(%)	471 (49.2)	4321 (43.1)	1309 (34.6)
Histology, *n *(%)			
Non-squamous	397 (41.4)	4094 (40.8)	1656 (43.8)
Adenocarcinoma	396 (41.3)	3958 (39.5)	1637 (43.3)
Squamous	370 (38.6)	4897 (48.8)	1640 (43.4)
NOS	169 (17.6)	886 (8.8)	358 (9.5)
Other	22 (2.3)	149 (1.5)	126 (3.3)
**Stage IIIC**^**b,c**^	***n* = 86 (1.2)**	***n* = 1167 (2.0)**	***n* = 1073 (5.2)**
Age, years			
Median (IQR)	67 (60–75)	70 (62–76)	67 (60–76)
Age ≥ 65 years, *n *(%)	53 (61.6)	810 (69.4)	659 (61.4)
Female, *n *(%)	42 (48.8)	468 (40.1)	332 (30.9)
Histology, *n *(%)			
Non-squamous	40 (46.5)	566 (48.5)	464 (43.2)
Adenocarcinoma	40 (46.5)	547 (46.9)	461 (43.0)
Squamous	40 (46.5)	493 (42.2)	474 (44.2)
NOS	PM	92 (7.9)	106 (9.9)
Other	PM	16 (1.4)	29 (2.7)

*AJCC* American Joint Committee on Cancer, *CAS* Cancer Analysis System, *IQR* Interquartile range, *NOS* Not otherwise specified, *NSCLC* Non-small cell lung cancer, *O2* Oncology Outcomes, *PM* Primary masked data, *TNM* Tumor, node, metastasis, *UICC* Union for International Cancer Control; *VONKOdb* Oncological Health Care Research Database

^a^Primary data masking was performed if patient counts for individual categories were between 1 and 9 for O2, between 1 and 5 for CAS, and between 1 and 4 for VONKOdb

^b^Proportions of patients diagnosed at each stage are based on the full analysis population at each data source. TNM stage was assigned at the date of NSCLC diagnosis. There were no restrictions on the source data used for TNM staging (clinical vs. post-surgical/pathological staging). For O2, TNM stage was derived only from clinical staging for this analysis. For CAS and VONKOdb, TNM stage could have been derived from either clinical staging or post-surgical/pathological staging

^c^Stage IIIC was introduced in the 8 th edition of the TNM classification system per the AJCC/UICC, which was officially utilized in the data sources from 2017/2018 onward

### Survival outcomes and conditional survival

**Fig. 3:**
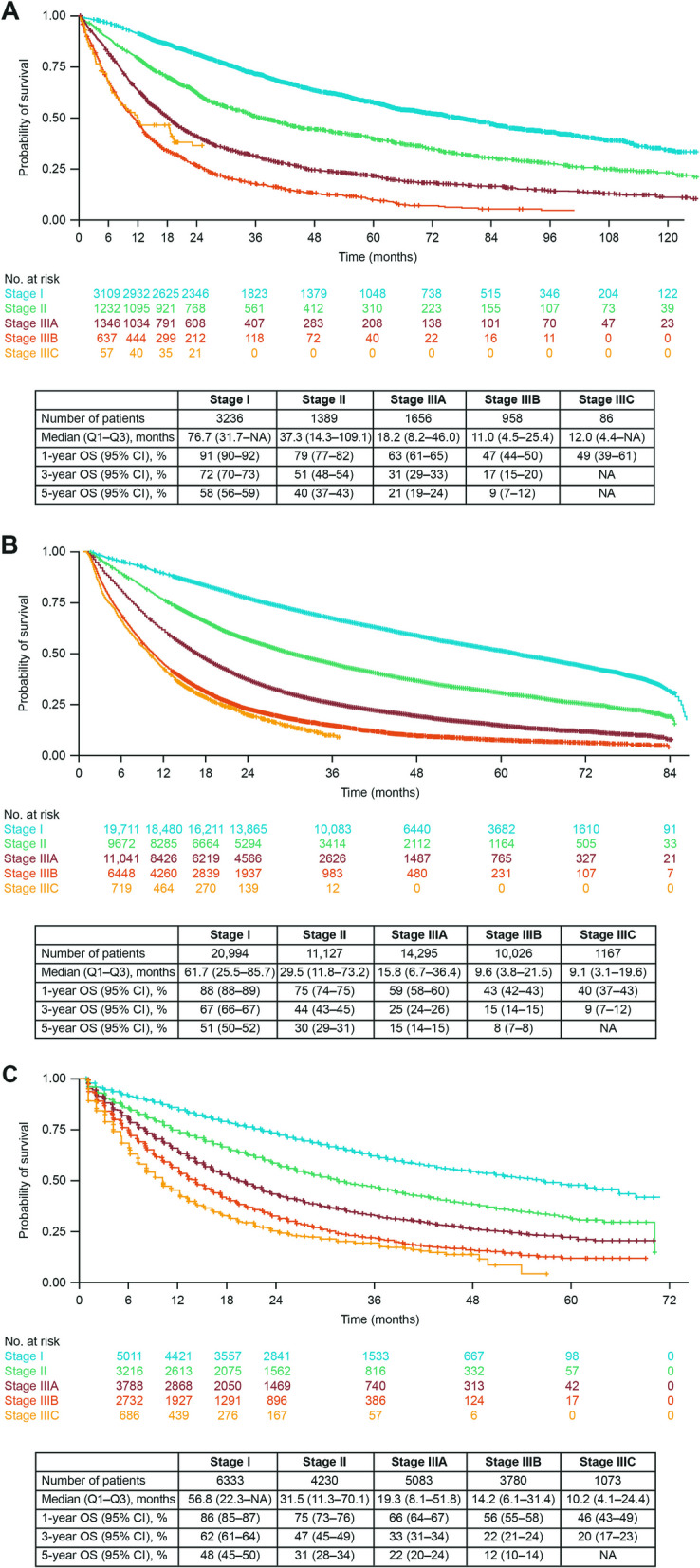
OS Kaplan–Meier curves for patients with stage I-IIIC NSCLC. **A** O2. **B** CAS. **C** VONKOdb. OS data are based on an index date of diagnosis. CAS, Cancer Analysis System; CI, confidence interval; NA, not available; NSCLC, non-small cell lung cancer; O2, Oncology Outcomes; OS, overall survival; Q, quartile; VONKOdb, Oncological Health Care Research Database

For all 3 data sources, median OS from diagnosis shortened, and landmark OS rates decreased with advancing disease stage through stage IIIB (Fig. 3). In patients with stage I and II NSCLC, median OS was longer for O2 (76.7 and 37.3 months, respectively) versus CAS (61.7 and 29.5 months) and VONKOdb (56.8 and 31.5 months), with OS rates showing the same trend. Among patients with stage IIIA or IIIB disease, median OS was slightly longer for VONKOdb (19.3 and 14.2 months, respectively) versus O2 (18.2 and 11.0 months) and CAS (15.8 and 9.6 months), with OS rates again showing the same trend. In patients with stage IIIC disease, OS outcomes for O2 were similar to those observed with stage IIIB, although the sample size was small; however, the trend for incrementally worse OS outcomes continued through stage IIIC for CAS and VONKOdb (Fig. 3).

In conditional survival analyses for all data sources, there was a trend toward an increased probability of surviving for a further 2 years after reaching sequential post-diagnosis survival landmarks among patients with stage II or III NSCLC (Table 2). In contrast, patients with stage I disease had a similar probability of surviving a further 2 years, regardless of the number of years they had already survived since their diagnosis (Table 2).

**Table 2:** Conditional OS probability by years survived from diagnosis

	**Probability (95% CI) of surviving a further 2 years**		
**Years survived from diagnosis**	**O2 (Canada)**	**CAS (England)**	**VONKOdb (Germany)**
**Stage I**^**a**^			
0	81 (80–83)	76 (76–77)	73 (72–75)
1	79 (77–80)	76 (75–76)	72 (71–74)
3	81 (79–83)	77 (76–78)	77 (73–80)
5	83 (80–85)	62 (58–66)	NA
**Stage II**^**a**^			
0	62 (60–65)	56 (55–57)	59 (57–60)
1	64 (61–67)	60 (58–61)	63 (61–65)
3	78 (75–82)	68 (67–70)	66 (61–72)
5	77 (72–82)	63 (57–70)	NA
**Stage IIIA**^**a**^			
0	41 (39–43)	36 (35–37)	43 (42–45)
1	49 (46–53)	43 (42–44)	50 (48–52)
3	70 (65–75)	58 (56–61)	67 (62–73)
5	76 (70–83)	54 (46–63)	NA
**Stage IIIB**^**a**^			
0	27 (24–30)	22 (21–23)	33 (31–35)
1	37 (33–42)	34 (33–36)	39 (37–42)
3	57 (48–68)	52 (49–56)	55 (47–64)
5	60 (45–79)	NA	NA
**Stage IIIC**^**a**^			
0	36 (27–49)	19 (17–22)	26 (23–29)
1	NA	23 (17–30)	43 (38–49)
3	NA	NA	NA
5	NA	NA	NA

NA denotes too few patients to conduct probability analysis or masked data

*CAS* Cancer Analysis System, *CI* Confidence interval, *NA* Not available, *O2* Oncology Outcomes, *OS* Overall survival, *VONKOdb* Oncological Health Care Research Database

^a^TNM stage was assigned at the date of NSCLC diagnosis. There were no restrictions on the source data used for TNM staging (clinical vs. post-surgical/pathological staging). For O2, TNM stage was derived only from clinical staging for this analysis. For CAS and VONKOdb, TNM stage could have been derived from either clinical staging or post-surgical/pathological staging

### Treatment patterns and associated survival outcomes

#### Treatment type (resected, unresected, or untreated/unclassified)

Across the different data sources and disease stages, 70.3–85.2% of patients received active treatment for their NSCLC. Proportions of patients who were resected versus unresected did not differ substantially for stages I (range, 58.9–66.9% vs. 11.7–19.8%, respectively) and II (range, 57.7–59.2% vs. 18.9–26.3%) (Table 3). However, through stages IIIA, IIIB, and IIIC, proportions of patients who were resected decreased (range, 24.7–42.7%, 4.6–21.8%, and 0.9–7.5%, respectively) in parallel with increased proportions who were unresected (range, 32.0–51.7%, 52.1–68.1%, and 62.7–82.6%) and there was a trend toward increased proportions categorized as untreated/unclassified (range, 21.6–27.1%, 26.1–27.3%, and 29.6–29.7%) (Table 4). Of note, while it was necessary to merge the untreated and unclassified initial treatment types because of masking rules preventing presentation of separate data, the proportion with an ‘unclassified’ treatment type was very low, regardless of stage or data source; across the data sources, proportions with an ‘unclassified’ treatment type ranged from a minimum of < 0.1% of patients with stage I or II NSCLC for CAS up to a maximum of 1.4% of patients with stage III disease for VONKOdb. While general trends were consistent across the data sources, proportions of patients with resected stage IIIA, IIIB, or IIIC disease were higher for VONKOdb (42.7%, 21.8%, and 7.5%, respectively) than for O2 (24.7%, 7.3%, and masked) or CAS (26.8%, 4.6%, and 0.9%) (Table 4).

**Table 3:** Initial treatment and associated median OS and survival rates for treated patients with stage I or II NSCLC from O2, CAS, and VONKOdb^a^

	**O2 (Canada)**				**CAS (England)**				**VONKOdb (Germany)**			
	**n (%)**	**Median OS (Q1-Q3), months**	**1-year OS, %**	**3-year OS, %**	**n (%)**	**Median OS (Q1-Q3), months**	**1-year OS, %**	**3-year OS, %**	**n (%)**	**Median OS (Q1-Q3), months**	**1-year OS, %**	**3-year OS, %**
**Stage I**^**b**^	***N* = 3236**				***N* = 20,994**				***N* = 6333**			
Resected^c^	1907 (58.9)				14,038 (66.9)				4092 (64.6)			
Surgery alone	1863 (97.7)	128.0 (59.8-NR)	96	85	13,326 (94.9)	80.3 (44.5-NR)	94	80	3903 (95.4)	NR (34.1-NR)	90	74
+ adj SACT	31 (1.6)	111.1 (32.4–116.1)	94	70	456 (3.2)	76.8 (36.4-NR)	94	75	117 (2.9)	NR (37.5-NR)	98	76
+ adj SACT + RT	PM	-	-	-	72 (0.5)	67.8 (15.4-NR)	86	62	18 (0.4)	36.1 (14.7-NR)	77	50
+ adj RT	PM	-	-	-	144 (1.0)	46.8 (20.1-NR)	83	55	32 (0.8)	29.3 (9.0-NR)	69	48
Unresected^c^	642 (19.8)				3859 (18.4)				742 (11.7)			
Concurrent CRT	PM	-	-	-	60 (1.6)	30.4 (16.2-NR)	87	46	12 (1.6)	23.4 (9.7–24.9)	67	22
RT alone	620 (96.6)	42.2 (18.0–75.2)	84	56	3544 (91.8)	30.8 (14.4–56.3)	79	43	587 (79.1)	24.4 (10.9–46.0)	73	33
SACT alone	PM	-	-	-	173 (4.5)	18.1 (8.3–34.8)	65	25	93 (12.5)	23.3 (7.8–40.6)	69	30
Untreated/unclassified^c,d^	687 (21.2)				3097 (14.8)				1499 (23.7)			
**Stage II**^**b**^	**N = 1389**				**N = 11,127**				**N = 4230**			
Resected^c^	802 (57.7)				6476 (58.2)				2502 (59.2)			
Surgery alone	494 (61.6)	69.1 (23.4–121.9)	87	67	3933 (60.7)	44.6 (18.1–82.6)	82	57	1578 (63.1)	38.5 (15.0–68.1)	78	53
+ adj SACT	272 (33.9)	NR (42.2-NR)	95	79	2041 (31.5)	73.4 (34.2-NR)	93	74	725 (29.0)	NR (35.3-NR)	91	74
+ adj SACT + RT	24 (3.0)	73.6 (14.9-NR)	79	62	283 (4.4)	38.8 (14.7-NR)	81	52	82 (3.3)	28.8 (13.0–60.3)	78	43
+ adj RT	10 (1.3)	32.9 (14.5-NR)	80	30	147 (2.3)	35.0 (15.0-NR)	81	49	48 (1.9)	30.3 (8.5-NR)	67	49
+ neoadj SACT	0	-	-	-	48 (0.7)	55.7 (25.0-NR)	90	64	35 (1.4)	NR (20.1-NR)	87	66
Unresected^c^	319 (23.0)				2924 (26.3)				799 (18.9)			
Concurrent CRT	15 (4.7)	68.1 (13.9–77.6)	80	60	237 (8.1)	25.4 (13.1–51.8)	80	35	32 (4.0)	29.4 (17.9-NR)	80	34
Sequential CRT	0	-	-	-	145 (5.0)	20.6 (11.5–38.3)	74	28	39 (4.9)	28.2 (8.2-NR)	67	48
Other curative SACT + RT	23 (7.2)	23.8 (11.1–29.5)	74	11	60 (2.1)	17.3 (11.6–50.4)	75	31	54 (6.8)	21.9 (9.4–32.5)	66	22
RT alone	263 (82.4)	16.4 (6.9–33.8)	59	24	2048 (70.0)	15.4 (7.1–30.7)	60	20	363 (45.4)	15.3 (5.6–33.5)	56	21
SACT alone	PM	-	-	-	311 (10.6)	15.0 (7.9–31.5)	60	22	270 (33.8)	27.4 (8.8-NR)	69	41
SACT + palliative RT	10 (3.1)	12.7 (7.9–28.0)	56	0	88 (3.0)	9.0 (5.4–17.8)	39	NA	22 (2.8)	12.0 (5.5–30.7)	55	11
SACT + RT (unknown intent)	0	-	-	-	35 (1.2)	12.9 (7.2-NR)	57	NA	16 (2.0)	27.8 (11.5-NR)	75	27
Untreated/unclassified^c,d^	268 (19.3)				1727 (15.5)				929 (22.0)			

*Adj* adjuvant, *CAS* Cancer Analysis System, *CRT* Chemoradiotherapy, *NA* Not available, *neoadj* neoadjuvant, *NR* Not reached, *NSCLC* Non-small cell lung cancer, *O2* Oncology Outcomes, *OS* Overall survival, *PM* Primary masked data, *Q* Quartile, *RT* Radiotherapy, *SACT* Systemic anticancer therapy, *VONKOdb* Oncological Health Care Research Database

^a^Initial treatment categories were algorithmically derived for each data source using a treatment decision algorithm developed as part of the I-O Optimise initiative as shown in Supplementary Fig. 1. Individual treatment categories were excluded from the table if data were masked and/or the number of patients recorded as receiving the treatment was < 30 for all three data sources. Median OS data are based on an index date of the start of treatment. Per specific data source requirements, individual categories including between 1 and 9 patients at O2, between 1 and 5 patients at CAS, and between 1 and 4 patients at VONKOdb underwent primary data masking

^b^TNM stage was assigned at the date of NSCLC diagnosis. There were no restrictions on the source data used for TNM staging (clinical vs. post-surgical/pathological staging). For O2, TNM stage was derived only from clinical staging for this analysis. For CAS and VONKOdb, TNM stage could have been derived from either clinical staging or post-surgical/pathological staging

^c^For each data source, proportions of patients who were resected, unresected, or untreated/unclassified are based on the total number of patients diagnosed at each stage; proportions receiving each specific treatment category are based on the total number of patients who were resected or unresected at each stage, as appropriate

^d^The untreated and unclassified initial treatment types were merged because of masking rules preventing presentation of separate data; the total number (%) of patients who were unclassified across stages I and II was < 10 (< 0.3%) for O2, < 12 (< 0.1%) for CAS, and 25 (0.2%) for VONKOdb

**Table 4:** Initial treatment and associated median OS and survival rates for treated patients with stage III NSCLC from O2, CAS, and VONKOdb^a^

	**O2 (Canada)**				**CAS (England)**					**VONKOdb (Germany)**			
	**n (%)**	**Median OS (Q1-Q3), months**	**1-year OS, %**	**3-year OS, %**	**n (%)**	**Median OS (Q1-Q3), months**	**1-year OS, %**	**3-year OS, %**	**n (%)**		**Median OS (Q1-Q3), months**	**1-year OS, %**	**3-year OS, %**
**Stage IIIA**^**b**^	***N* = 1656**				***N* = 14,295**				***N* = 5083**				
Resected^c^	409 (24.7)				3825 (26.8)				2172 (42.7)				
Surgery alone	178 (43.5)	35.8 (14.5–98.3)	76	50	1727 (45.2)	23.5 (9.4–54.2)	70	36	976 (44.9)		22.9 (8.1–53.0)	68	37
+ adj SACT	150 (36.7)	48.0 (19.4–128.3)	89	63	1385 (36.2)	46.4 (20.9-NR)	88	59	547 (25.2)		42.5 (20.2-NR)	86	57
+ adj SACT + RT	31 (7.6)	58.8 (13.8-NR)	77	58	455 (11.9)	30.1 (14.0–64.7)	80	46	290 (13.4)		45.5 (19.2-NR)	87	56
+ adj RT	20 (4.9)	8.4 (4.0–18.8)	40	10	93 (2.4)	17.9 (5.8–39.7)	61	32	95 (4.4)		18.3 (10.0–45.9)	66	30
+ neoadj SACT	PM	-	-	-	146 (3.8)	46.4 (19.8-NR)	90	59	146 (6.7)		51.8 (14.5-NR)	78	55
+ neoadj SACT + RT	17 (4.2)	53.9 (41.9-NR)	94	87	8 (0.2)	-	-	-	49 (2.3)		19.3 (10.1-NR)	69	35
+ perioperative SACT	PM	-	-	-	SM	-	-	-	55 (2.5)		NR (14.5-NR)	84	51
Unresected^c^	798 (48.2)				7389 (51.7)				1626 (32.0)				
Concurrent CRT	125 (15.7)	42.8 (15.9-NR)	83	55	1286 (17.4)	26.5 (13.3–58.3)	78	40	115 (7.1)		28.5 (12.7-NR)	77	40
Sequential CRT	PM	-	-	-	751 (10.2)	19.7 (10.9–38.1)	72	27	171 (10.5)		13.6 (7.4–42.3)	55	29
Other curative SACT + RT	139 (17.4)	28.5 (12.2–67.3)	77	38	262 (3.5)	21.8 (11.6–51.3)	73	30	158 (9.7)		24.3 (10.5-NR)	71	30
RT alone	390 (48.9)	9.9 (4.6–22.0)	42	14	2958 (40.0)	10.4 (4.3–22.3)	46	14	391 (24.0)		11.7 (5.1–24.5)	49	18
SACT alone	72 (9.0)	15.8 (8.0–33.4)	60	21	1426 (19.3)	12.7 (6.0–27.0)	52	18	601 (37.0)		13.4 (5.7–33.8)	55	24
SACT + palliative RT	69 (8.6)	12.4 (5.5–24.8)	50	16	536 (7.3)	9.1 (5.6–17.1)	39	9	102 (6.3)		8.8 (4.7–17.8)	37	13
SACT + RT (unknown intent)	0	-	-	-	SM	17.2 (9.1–33.2)	67	23	76 (4.7)		22.9 (11.9–50.8)	74	36
Untreated/unclassified^c,d^	449 (27.1)				3081 (21.6)				1285 (25.3)				
**Stage IIIB**^**b**^	***N* = 958**				***N* = 10,026**				**N = 3780**				
Resected^c^	70 (7.3)				460 (4.6)				824 (21.8)				
Surgery alone	19 (27.1)	25.5 (11.5–112.6)	73	32	SM	13.3 (5.3–38.7)	51	27	283 (34.3)		12.1 (3.1–30.1)	50	19
+ adj SACT	19 (27.1)	39.6 (23.0–44.4)	95	67	SM	29.6 (17.5-NR)	84	42	129 (15.7)		18.6 (8.6–45.6)	66	34
+ adj SACT + RT	PM	-	-	-	SM	21.0 (12.5-NR)	78	NA	196 (23.8)		27.3 (13.3-NR)	77	40
+ adj RT	15 (21.4)	5.6 (3.2–18.9)	40	13	9 (2.0)	-	-	-	39 (4.7)		12.2 (7.6–31.5)	53	15
+ neoadj SACT	PM	-	-	-	SM	-	-	-	101 (12.3)		28.9 (11.0-NR)	70	45
+ neoadj SACT + RT	PM	-	-	-	PM	-	-	-	31 (3.8)		20.6 (7.1-NR)	62	27
+ perioperative SACT	0	-	-	-	PM	-	-	-	39 (4.7)		30.3 (7.5-NR)	68	38
Unresected^c^	629 (65.7)				6827 (68.1)				1969 (52.1)				
Concurrent CRT	49 (7.8)	30.8 (11.0–59.0)	69	46	845 (12.4)	23.7 (10.8–52.6)	72	36	163 (8.3)		20.9 (9.7–43.8)	67	29
Sequential CRT	PM	-	-	-	668 (9.8)	18.3 (10.6–36.7)	70	26	198 (10.1)		19.4 (9.0–57.9)	66	29
Other curative SACT + RT	50 (7.9)	27.5 (17.2–55.8)	78	37	174 (2.5)	19.4 (12.0–33.0)	75	24	193 (9.8)		22.5 (10.0–40.2)	68	25
RT alone	327 (52.0)	6.5 (2.9–14.9)	30	8	2050 (30.0)	5.7 (2.2–12.9)	27	7	359 (18.2)		10.2 (4.0–21.4)	45	13
SACT alone	107 (17.0)	18.0 (9.5–41.1)	67	28	2081 (30.5)	12.2 (5.1–26.1)	51	17	730 (37.1)		12.0 (4.4–27.4)	50	19
SACT + palliative RT	95 (15.1)	10.9 (4.7–24.8)	45	15	858 (12.6)	8.9 (5.6–16.9)	37	10	201 (10.2)		11.3 (4.8–27.9)	47	21
SACT + RT (unknown intent)	SM	-	-	-	SM	15.2 (6.2–27.0)	56	18	97 (4.9)		21.3 (8.0-NR)	66	31
Untreated/unclassified^c,d^	259 (27.0)				2739 (27.3)				987 (26.1)				
**Stage IIIC**^**b**^	**N = 86**				**N = 1167**				**N = 1073**				
Resected^c^	PM				11 (0.9)				81 (7.5)				
Unresected^c^	71 (82.6)				811 (69.5)				673 (62.7)				
Concurrent CRT	PM	-	-	-	72 (8.9)	20.3 (9.3-NR)	65	0	58 (8.6)		15.5 (7.1–52.0)	61	30
Sequential CRT	0	-	-	-	61 (7.5)	19.9 (11.9-NR)	74	0	58 (8.6)		14.3 (7.5–27.6)	59	22
Other curative SACT + RT	PM	-	-	-	11 (1.4)	NR (NR-NR)	NA	0	57 (8.5)		14.8 (6.3–35.8)	56	17
RT alone	27 (38.0)	5.5 (1.3–11.7)	25	9	172 (21.2)	4.8 (2.0–10.5)	19	0	108 (16.0)		5.5 (3.4–17.5)	37	7
SACT alone	21 (29.6)	NR (14.5-NR)	76	56	366 (45.1)	14.4 (6.2–26.0)	55	NA	258 (38.3)		10.1 (4.0–29.3)	43	20
SACT + palliative RT	17 (23.9)	21.2 (4.9–35.6)	65	24	107 (13.2)	8.6 (5.3–15.2)	32	0	86 (12.8)		10.6 (5.3–23.6)	43	20
SACT + RT (unknown intent)	0	-	-	-	22 (2.7)	15.3 (8.9-NR)	55	0	35 (5.2)		18.0 (9.2-NR)	69	29
Untreated/unclassified^c,d^	SM				345 (29.6)				319 (29.7)				

*Adj* Adjuvant, *CAS* Cancer Analysis System, *CRT* Chemoradiotherapy, *NA* Not available, *neoadj* neoadjuvant, *NR* Not reached, *NSCLC* Non-small cell lung cancer, *O2* Oncology Outcomes, *OS* Overall survival, *PM* Primary masked data, *Q* Quartile, *RT* Radiotherapy, *SACT* Systemic anticancer therapy, *SM* Secondary masked data, *VONKOdb* Oncological Health Care Research Database

^a^Initial treatment categories were algorithmically derived for each data source using a treatment decision algorithm developed as part of the I-O Optimise initiative as shown in Supplementary Fig. 1. Individual treatment categories were excluded from the table if data were masked and/or the number of patients recorded as receiving the treatment was < 30 for all three data sources. Median OS data are based on an index date of the start of treatment. Per specific data source requirements, individual categories including between 1 and 9 patients at O2, between 1 and 5 patients at CAS, and between 1 and 4 patients at VONKOdb underwent primary data masking. Additional secondary data masking was performed where necessary to prevent unmasking of categories undergoing primary data masking

^b^TNM stage was assigned at the date of NSCLC diagnosis. There were no restrictions on the source data used for TNM staging (clinical vs. post-surgical/pathological staging). For O2, TNM stage was derived only from clinical staging for this analysis. For CAS and VONKOdb, TNM stage could have been derived from either clinical staging or post-surgical/pathological staging

^c^For each data source, proportions of patients who were resected, unresected, or untreated/unclassified are based on the total number of patients diagnosed at each stage; proportions receiving each specific treatment category are based on the total number of patients who were resected or unresected at each stage, as appropriate

^d^The untreated and unclassified initial treatment types were merged because of masking rules preventing presentation of separate data; the total number (%) of patients who were unclassified across stages IIIA, IIIB, and IIIC was 22 (0.8%) for O2, < 19 (< 0.1%) for CAS, and 142 (1.4%) for VONKOdb

#### Patterns of initial treatment and survival for stage I or II NSCLC

Across the data sources, most patients with resected stage I NSCLC received surgery alone (range, 94.9−97.7%), with the reached median OS ranging from 80.3 to 128.0 months (median OS for these patients was not reached [NR] at VONKOdb, with a lower interquartile range [IQR] value of 34.1 months), and 3-year OS ranging from 74 to 85% (Table 3). Likewise, most patients with resected stage II disease received surgery alone (range, 60.7–63.1%), with median OS ranging from 38.5 to 69.1 months, and 3-year OS ranging from 53 to 67%. Around one-third of patients with resected stage II disease received adjuvant SACT alone or with RT (SACT ± RT) (range, 32.3–36.9%), with the reached median OS ranging from 28.8 to 73.6 months (median OS for patients receiving adjuvant SACT alone was NR at O2 and VONKOdb, with lower IQR values of 42.2 and 35.3 months, respectively), and 3-year OS ranging from 43 to 79%. The proportion of patients with resected stage I or II NSCLC who received neoadjuvant SACT ± RT was uniformly low; data were masked for O2 and proportions across the CAS and VONKOdb data sources did not exceed 1.4%.

Among patients with unresected stage I NSCLC, most received RT alone (range, 79.1–96.6%), with median OS ranging from 24.4 to 42.2 months, and 3-year OS ranging from 33 to 56% (Table 3). In patients with unresected stage II disease, treatment allocation varied by data source. Most patients received RT alone for O2 (82.4%) and CAS (70.0%), with respective median OS of 16.4 and 15.4 months, and 3-year OS of 24% and 20%. Data on patients receiving SACT alone was masked at O2, while 10.6% of patients at CAS received SACT alone, with a median OS of 15.0 months and a 3-year OS of 22%. For VONKOdb, 45.4% received RT alone, with a median OS of 15.3 months and 3-year OS of 21%, and 33.8% received SACT alone, with a median OS of 27.4 months and 3-year OS of 41% (Table 3).

#### Patterns of initial treatment and survival for stage IIIA-C NSCLC

Across the data sources, most patients with resected stage IIIA NSCLC received surgery alone (range, 43.5–45.2%) or with adjuvant SACT ± RT (range, 38.5–48.1%); median OS and 3-year OS ranged from 22.9 to 35.8 months and 36 to 50%, respectively, with surgery alone, and from 30.1 to 58.8 months and 46 to 63% with surgery with adjuvant SACT ± RT (Table 4). Assessing patterns of initial treatment and survival among patients with resected stage IIIB or IIIC disease was impacted by small sample sizes and/or data masking. However, for VONKOdb, 34.3% of patients with resected stage IIIB disease received surgery alone, with a median OS of 12.1 months and 3-year OS of 19%, and 39.4% received surgery with adjuvant SACT ± RT, with a median OS range of 18.6–27.3 months and a 3-year OS range of 34–40% (Table 4). The proportion of patients with resected stage IIIA-C NSCLC who received neoadjuvant SACT ± RT was relatively low across the data sources, with the noteworthy exception that 12.3% of patients with resected stage IIIB disease for VONKOdb received neoadjuvant SACT, with a corresponding median OS of 28.9 months and 3-year OS of 45% (Table 4).

Among patients with unresected stage IIIA-C NSCLC, the majority received either RT alone, SACT alone, or SACT + palliative RT, with variations by data source (Table 4). For O2, these treatments were received by 66.5% of patients with unresected stage IIIA (with a median OS range of 9.9–15.8 months and 3-year OS range of 14–21%), 84.1% with unresected stage IIIB (with a median OS range of 6.5–18.0 months and 3-year OS range of 8–28%), and 91.5% with unresected stage IIIC disease (with a reached median OS range of 4.8–21.2 months [median OS for patients receiving SACT alone at O2 was NR with a lower IQR value of 14.5 months] and 3-year OS range of 9–56%). For CAS, these treatments were received by 66.6% of patients with unresected stage IIIA (with a median OS range of 9.1–12.7 months and 3-year OS range of 9–18%), 73.1% with unresected stage IIIB (with a median OS range of 5.7–12.2 months and 3-year OS range of 7–17%), and 79.5% with unresected stage IIIC disease (with a median OS range of 4.8–14.4 months). For VONKOdb, these treatments were received by 67.3% of patients with unresected stage IIIA (with a median OS range of 8.8–13.4 months and 3-year OS range of 13–24%), 65.5% with unresected stage IIIB (with a median OS range of 10.2–12.0 months and 3-year OS range of 13–21%), and 67.2% with unresected stage IIIC disease (with a median OS range of 5.5–10.6 months and 3-year OS range of 7–20%). Of these treatments, RT alone and SACT alone were the most commonly administered, regardless of stage. However, the proportions of patients with unresected stage IIIA-C disease receiving SACT + palliative RT increased with increasing disease stage for all data sources (Table 4).

Across the data sources, a sizable minority of patients with unresected stage IIIA-C NSCLC received CRT (concurrent or sequential) or other curative SACT + RT (Table 4). Among patients with unresected stage IIIA and IIIB NSCLC for O2, concurrent CRT was received by 15.7% (with a median OS of 42.8 months and 3-year OS of 55%) and 7.8% (with a median OS of 30.8 months and 3-year OS of 46%), respectively; respective proportions receiving other curative SACT + RT were 17.4% (with a median OS of 28.5 months and 3-year OS of 38%) and 7.9% (with a median OS of 27.5 months and 3-year OS of 37%). Among patients with unresected stage IIIA, IIIB, and IIIC NSCLC for CAS, either concurrent or sequential CRT was received by 27.6% (with a median OS range of 19.7–26.5 months and 3-year OS range of 27–40%), 22.2% (with a median OS range of 18.3–23.7 months and 3-year OS range of 26–36%), and 16.4% (with a median OS range of 19.9–20.3 months and a 3-year OS of 0%), respectively; proportions receiving other curative SACT + RT were low for CAS, with only 3.5%, 2.5%, and 1.4% of patients with unresected stage IIIA, IIIB, and IIIC receiving this initial treatment, respectively. Among patients with unresected stage IIIA, IIIB, and IIIC NSCLC for VONKOdb, concurrent or sequential CRT was received by 17.6% (with a median OS range of 13.6–28.5 months and 3-year OS range of 29–40%), 18.3% (with a median OS range of 19.4–20.9 months and 3-year OS of 29%), and 17.2% (with a median OS range of 14.3–15.5 months and 3-year OS range of 22–30%), respectively; respective proportions receiving other curative SACT + RT were 9.7% (with a median OS of 24.3 months and 3-year OS of 30%), 9.8% (with a median OS of 22.5 months and 3-year OS of 25%), and 8.5% (with a median OS of 14.8 months and 3-year OS of 17%) (Table 4).

Only a small number of patients across the data sources were categorized as receiving concurrent CRT in combination with immunotherapy based on our algorithmic definition. For O2, no patients received this treatment at any stage; for CAS, a small number of patients with stage IIIA and IIIB NSCLC received this treatment, with associated data masking; and for VONKOdb, this treatment was received by < 1.0% of patients with unresected stage II or IIIA, 1.4% of patients with unresected stage IIIB, and 1.9% of those with unresected stage IIIC disease. Respective median OS and 3-year OS were not available for stage II, were NR (with a lower IQR value of 21.5 months) and 62%, for stage IIIA, 24.0 months and 42% for stage IIIB, and 23.2 months and 41% for stage IIIC.

## Discussion

The recent emergence of new therapies and treatment strategies for non-metastatic NSCLC has resulted in a rapid evolution of the associated treatment landscape. In this fast-moving environment, it is important to establish real-world baselines (i.e., based on the period before the emergence of newer therapies) against which future changes in patient management and clinical outcomes can be compared, allowing accurate measurement of the effect of these new treatment approaches. Our study provides such a baseline, presenting patient characteristics, treatment patterns, and survival outcomes before the widespread availability of newer immune checkpoint inhibitors and tyrosine kinase inhibitors for more than 85,000 patients diagnosed with non-metastatic NSCLC across Canada, England, and Germany, between 2010 and 2020.

Study populations for the data sources showed variations in patient age, sex, and NSCLC histology. However, the most noteworthy differences were in the distribution of disease stage at diagnosis, with the proportion of patients diagnosed at stage I higher for O2 and the proportion of patients diagnosed at stage III higher for VONKOdb. There are various factors that could have contributed to these observations, including inherent differences in country-specific healthcare systems (e.g., access to care, primary care referral procedures, extent of public versus private cancer services, etc.) and the racial and socioeconomic make-up of the respective populations [42–46]. Furthermore, screening-related factors could have also contributed to the observed higher proportion of patients diagnosed at stage I for O2. Indeed, although dedicated lung cancer screening programs were not formally established in Alberta until 2024, meaning they would not have impacted diagnostic patterns in this study, the Canadian Task Force on Preventive Health Care released general recommendations on lung cancer screening in 2016 [47] that might have led to early uptake of “screening-like” behaviors among Canadian physicians. Moreover, the Alberta Thoracic Oncology Program, an automatic referral process for patients with chest computed tomographic scans suggestive of malignant disease, was established in 2011, primarily in response to unacceptably long wait times for lung cancer diagnosis and treatment [48], and could also have influenced the stage distribution at O2 in this study. Also, as several studies have shown a level of stage migration across all non-metastatic NSCLC stages between the 7 th and 8 th editions of the TNM classification systems [49–51], the relative proportions diagnosed at each stage for each data source could also be influenced in part by the specific study inclusion period and the edition of the TNM classification system used during this period.

OS data for the full study populations from the data sources showed similar trends, with OS outcomes generally declining with increasing disease stage, as expected. Furthermore, the OS outcomes observed in the current study were generally aligned with those reported elsewhere for similar real-world patient populations from Denmark, Portugal, and Sweden [6, 28, 52]. In addition, although the O2 data source is restricted to the province of Alberta, the OS data from O2 aligned closely with those reported for patients with lung and bronchus cancer in a population-based study of cancer survival across Canada, excluding Quebec, between 2010 and 2017 [53], which suggests that the O2 results are generally representative of Canada overall. In the current study, numerical differences in OS outcomes between the data sources were noted, particularly in relation to the apparent better outcomes observed among patients with stage I or II NSCLC in Canada versus England and Germany. However, this observed difference could have been influenced by a multitude of factors, most crucially, the prognosis of the patients and associated distribution of initial treatment (as will be discussed later). Other factors that might have also influenced OS across the data sources include the observed differences in patient/disease characteristics (e.g., aforementioned differences in age, sex, and histology distribution), as well as potential variations in other characteristics that were only partially available, such as patient performance status (only available for CAS) and comorbidity status (only available for O2), which have both been shown to be potentially prognostic in early-stage NSCLC [54–58]. Finally, OS outcomes could also have been influenced by country-specific approaches to the subsequent treatment (i.e., the respective availability of immunotherapy-based and targeted therapies) and palliative care of patients as they progress to the advanced stages of lung cancer, as well as the underlying general mortality in each country.

Although there was some variability in the relative proportions receiving specific initial treatments in the different data sources, trends in treatment allocation were generally aligned with relevant European and North American lung cancer guidelines for early-stage and locally advanced NSCLC that were available during the respective study periods [9, 10, 12, 14, 16, 59]. As expected, surgical approaches were generally the mainstay of treatment for patients with stage I and II NSCLC. In these patients, those undergoing surgery alone or with (neo)adjuvant SACT ± RT achieved better OS outcomes compared with those receiving nonsurgical treatment (mostly with RT alone) across all three data sources. While this may suggest an advantage of surgical intervention in early-stage NSCLC, it is important to consider that both the decision on the most appropriate initial treatment and the subsequent treatment-related outcomes would likely have been influenced by differences in some of the aforementioned known or partially known patient characteristics, such as age, performance status, or comorbidity status (e.g., patients undergoing surgery may have been younger and fitter/healthier than those not selected for surgery, which could have impacted the respective survival outcomes). Among patients with stage III NSCLC, the majority received only nonsurgical treatments, and most patients with unresected stage III disease received (presumably palliative) treatment with SACT or RT alone or treatment with SACT + palliative RT. Interestingly, OS outcomes in patients with resected stage III NSCLC receiving (neo)adjuvant SACT ± RT were relatively similar to those achieved among patients with unresected stage III disease receiving curative CRT (concurrent or sequential). In addition, among the stage III population, the proportion of patients receiving surgery was notably higher for VONKOdb than O2 and CAS. This suggests a potentially increased preference for surgical management of stage III tumors among treating physicians in Germany versus Canada or England, which may have been influenced by the fact that the German stage III population was generally younger than the stage III populations in Canada and England (see Table 1) and typically had less nodal involvement than those in Canada and England (see Supplementary Table 3). It should also be noted that this observation may also have been influenced by potential differences in other factors that impact treatment decisions, but which were only partially available from the participating data sources (e.g., performance or comorbidity status) as well as possible differential access to specialist cancer care across the data sources (e.g., patients presenting at a tertiary center may be more likely to be referred for surgery than those presenting at community sites). Across the data sources and disease stages, around 70–80% of patients received active treatment, with a trend toward lower proportions among those with stage III disease.

Another important observation from this study was that, despite an expected variability across the data sources, OS outcomes in these studies appeared to be generally aligned with those reported elsewhere. For example, in the Lung Adjuvant Cisplatin Evaluation group pooled analysis of trials of patients with resected, primarily stage II-III NSCLC receiving adjuvant chemotherapy, median OS was approximately 56 months, with a 3-year OS of approximately 60% [60]; here, the range of reached median OS for patients with resected stage II-IIIA disease who received adjuvant SACT was 42.5–73.4 months, with a 3-year OS range of 57–79%. Similarly, in a recent study of perioperative pembrolizumab, median OS for patients with resected stage II-IIIB NSCLC in the placebo arm (i.e., those receiving neoadjuvant chemotherapy only) was 45.5 months, with a 3-year OS of approximately 60% [61]; here, the range of reached median OS for patients with resected stage II-IIIB disease who received neoadjuvant SACT was 28.9–55.7 months, with a 3-year OS range of 45–66% [61]. Likewise, in the PACIFIC study of durvalumab after concurrent CRT for unresectable stage IIIA-B NSCLC, median OS for the placebo arm (i.e., patients receiving concurrent CRT only) was 29.1 months, with a 3-year OS of 43.6% [62]; here, the range of median OS for patients with unresected stage IIIA-C disease who received concurrent CRT was 15.5–42.8 months, with a 3-year OS range of 29–55%. Interestingly, the 3-year OS range for a small group of patients with unresected stage IIIA-C disease who received concurrent CRT with immunotherapy in this analysis (41–62%) was relatively aligned with that reported for patients receiving concurrent CRT with durvalumab in the PACIFIC study (3-year OS of 56.7%) [62].

The study populations providing data for this analysis would be considered generally representative of an unselected non-metastatic NSCLC population, with the primary reason for the limited study attrition being missing information (for O2 and CAS, this was primarily missing sub-staging information; for VONKOdb, this was primarily missing follow-up information). Nevertheless, it is important to acknowledge that the current study represents a retrospective and purely descriptive analysis across distinct data sources and was not prospectively designed to compare between the patient populations. As such, any differences between the data sources noted in this article are purely observational. Furthermore, there are several other potentially limiting factors that should be considered when interpreting the results of this analysis. First, the inclusion and follow-up periods differed between the data sources and between the regional, population-based registries included in VONKOdb, which is relevant to the TNM classification system used, and may have influenced some of the noted observations. Second, due to their individualized “structures”, the data sources may have differed from one another in terms of their coverage of patients receiving care at tertiary versus non-tertiary centers, with associated differences in the availability of treatment that could potentially influence the observed patterns of initial treatment. Third, as there is an inherent potential for some missing/incomplete treatment data in real-word databases, and because of the use of algorithmically derived initial treatment categories, there was also potential for a limited amount of misclassification between these categories that might have influenced differences in treatment patterns across the participating data sources (this aspect is being actively addressed within the I-O Optimise initiative through continued modification and optimization of the developed algorithm). Fourth, small numbers of patients receiving certain initial treatments (and/or the associated primary or secondary data masking) limited interpretation of some of the OS outcomes data and impacted assessment of the relative effectiveness of those treatments within each data source. Fifth, the reported OS outcomes for initial treatment categories are influenced by immortal time bias, where patients are considered immortal between the start of the first and start of the last event in the respective treatment sequence; durations of treatment also varied between treatment categories, and therefore the amount of immortal time bias varies between categories. Sixth, considering literature indicating a level of discordance between clinical and post-surgical/pathological staging and highlighting potentially associated challenges in determining tumor resectability [63–65], the use of either clinical or post-surgical/pathological staging in the current study may have influenced treatment selection, as well as the observed OS outcomes in patients receiving surgery-based versus non-surgical treatment approaches. Moreover, in view of the emergence of various neoadjuvant chemo-immunotherapy options for non-metastatic NSCLC [66, 67], the use of either clinical or post-surgical/pathological staging for this analysis should be an important consideration when comparing outcomes from this baseline dataset with those from future studies of newer treatment strategies. Seventh and finally, since the study follow-up periods for all the data sources overlapped in part with the COVID-19 pandemic, there is the potential that this may have impacted treatment decisions and/or disrupted patient treatment schedules.

## Conclusion

These data from a large population of patients diagnosed with non-metastatic NSCLC in Canada, England, and Germany, provide useful insights into patient characteristics, treatment patterns, and survival outcomes before the widespread use of immunotherapy-based and targeted therapies. With the continued collection of data in the O2, CAS, and VONKOdb data sources, these data represent an important baseline and will allow for future evaluation of the real-world impact of the emerging treatment options for patients with non-metastatic NSCLC.

## Supplementary Information

Supplementary Material 1.

## Data Availability

The data from this study are not publicly available, and no data sharing is planned. Patient-level data cannot be shared due to regulatory and confidentiality reasons. Further questions on data sharing should be directed to the corresponding author (Alastair Greystoke).

## Abbreviations

Adj: Adjuvant
AJCC: American Joint Committee on Cancer
CAS: Cancer Analysis System
CI: Confidence interval
CRT: Chemoradiotherapy
EGFR: Epidermal growth factor receptor
ICD-10: International Classification of Diseases and Related Health Problems, 10th Revision
ICD-O-2: International Classification of Diseases for Oncology, 2nd edition
ICD-O-3: International Classification of Diseases for Oncology, 3rd edition
IQR: Interquartile range
NA: Not available
Neoadj: Neoadjuvant
NHS: National Health Service
NOS: Not otherwise specified
NR: Not reached
NSCLC: Non-small cell lung cancer
O2: Oncology Outcomes
OS: Overall survival
PD-1: Programmed death 1
PD-L1: Programmed death ligand 1
PM: Primary masked data
Q: Quartile
RT: Radiotherapy
SACT: Systemic anticancer therapy
SM: Secondary masked data
TNM: Tumor, node, metastasis
UICC: Union for International Cancer Control
VONKOdb: Oncological Health Care Research Database
