# Supplementary material for: Real-world treatment patterns and outcomes for patients with non-metastatic non-small cell lung cancer: retrospective analyses in Canada, England, and Germany
Source: BMC Pulm Med. 2025 May 27;25:265. doi: 10.1186/s12890-025-03715-9 (PMC12107783; doi:10.1186/s12890-025-03715-9)
Supplement: Supplementary file 1 — Supplementary Material 1. [file 12890_2025_3715_MOESM1_ESM.docx]

**SUPPLEMENT**

# Supplementary Methods: Participating Data Sources

## Oncology Outcomes (O2) – Canada

The O2 database, in partnership with Alberta Health Services and Cancer Care Alberta, collects cancer-related data for the province of Alberta in Canada, representing approximately 4.5 million residents. O2 is a multi-source database, integrating registry, electronic medical record, administrative, claims, and pharmacy data from 17 cancer centers (two tertiary centers, four regional centers, and 11 community sites). The database is primarily based on a dataset derived from the Alberta Cancer Registry, which is responsible for collating data on all new primary cancers, as well as all cancer-related deaths occurring in Alberta. Within this database, the Alberta Cancer Registry dataset is linked to other relevant datasets (e.g., Alberta Vital Statistics).

## Cancer Analysis System (CAS) – England

The CAS registry is a national database held in the National Cancer Registration and Analysis System area of Public Health England, which captures data on all cancer cases where medical care is provided by the National Health Service among the population of England (approximately 55 million people during the inclusion period of this study [1]. The CAS database holds up to 1000 data items for each patient with cancer, including detailed information on patient demographics, tumor staging, treatment, and hospital use. Data on deaths are incorporated into the CAS registry through linkage to the United Kingdom’s National Death Registry [2, 3].

## Oncological Health Care Research Database (VONKOdb) – Germany

The Oncological Health Care Research Database (VONKOdb) was established by a research group from the Institute of Social Medicine and Epidemiology at the University of Lübeck, Lübeck, Germany, and is a cancer-related database comprising pooled data from four regional, population-based clinical cancer registries for the federal states of Hamburg, Baden-Württemberg, North Rhine-Westphalia, and Schleswig-Holstein, representing approximately 33 million residents and approximately 40% of the population of Germany [4]. Based on the German Early Cancer Detection and Registration Act (Krebsfrüherkennungs- und -registergesetz) of 2013, and in accordance with the Association of German Tumor Centers and the Association of Population-Based Cancer Registries in Germany, all four federal state registries are required to collect a standardized “oncological core dataset” (Onkologischer Basisdatensatz). This dataset covers all types of cancer and is continuously supplemented by tumor-specific modules, with data on patient demographics, treatment patterns, and clinical outcomes extensively captured.

# Supplementary Table 1 Lung cancer ICD 10 diagnostic codes used for study inclusion

| ICD-10 code | Description | Data source |
| --- | --- | --- |
| C33 | Malignant neoplasm of trachea | O2 and CAS |
| C34.0 | Malignant neoplasm: Main bronchus (carina, hilus of lung) | O2, CAS, and VONKOdb |
| C34.1 | Malignant neoplasm: Upper lobe, bronchus or lung | O2, CAS, and VONKOdb |
| C34.2 | Malignant neoplasm: Middle lobe, bronchus or lung | O2, CAS, and VONKOdb |
| C34.3 | Malignant neoplasm: Lower lobe, bronchus or lung | O2, CAS, and VONKOdb |
| C34.8 | Malignant neoplasm: Overlapping lesion of bronchus or lung | O2, CAS, and VONKOdb |
| C34.9 | Malignant neoplasm: Unspecified part of bronchus or lung | O2, CAS, and VONKOdb |

CAS, Cancer Analysis System; ICD-10, International Classification of Diseases and Related Health Problems, 10th Revision; O2, Oncology Outcomes; VONKOdb, Oncological Health Care Research Database.

# Supplementary Fig. 1 Initial treatment algorithm


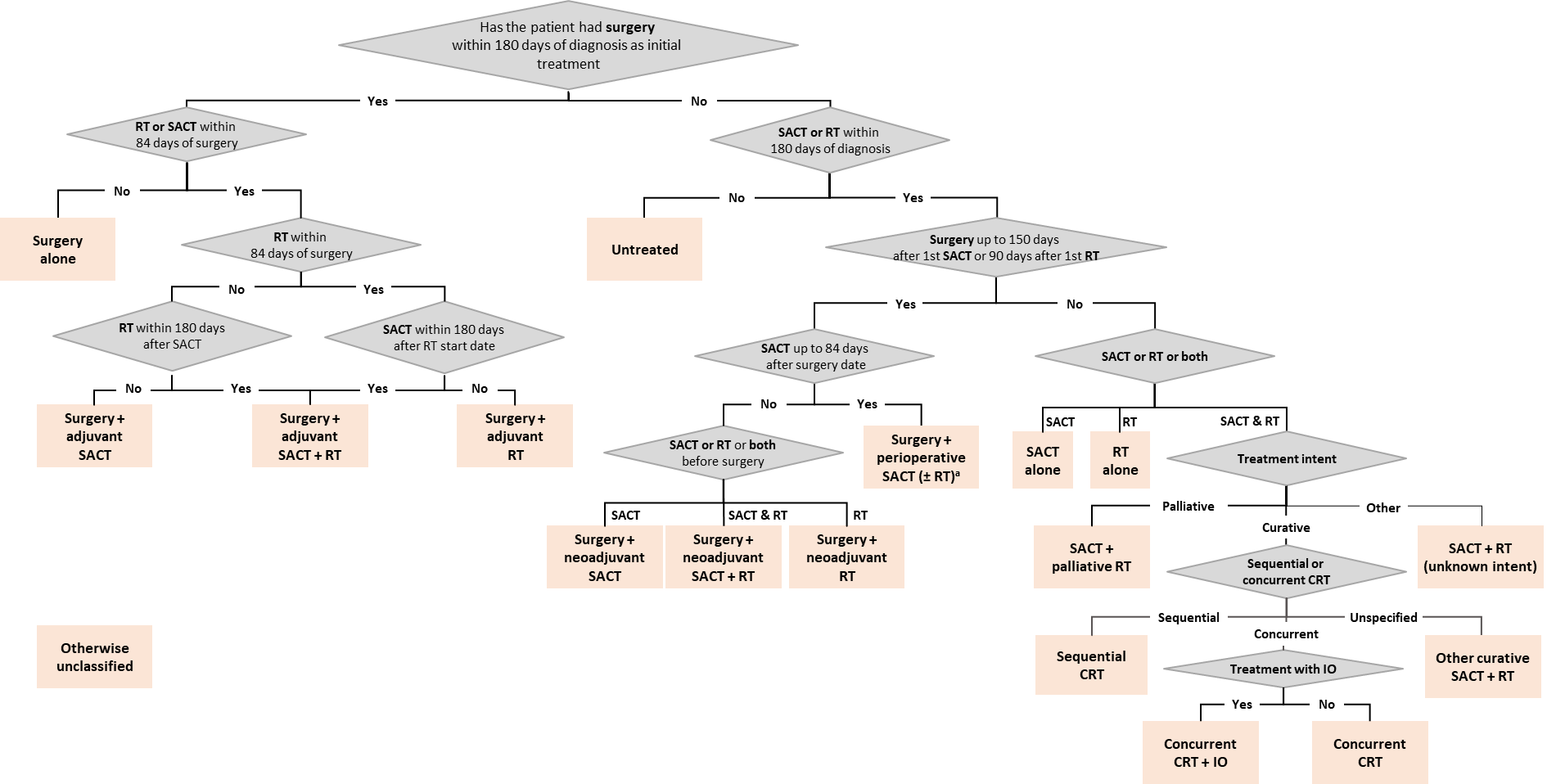


CAS, Cancer Analysis System; CRT, chemoradiotherapy; IO, immunotherapy; O2, Oncology Outcomes; RT, radiotherapy; SACT, systemic anticancer therapy; VONKOdb, Oncological Health Care Research Database.

^a^ As described in **Supplementary Table 2**, this category (i) must include administration of SACT before surgery, and (ii) allows administration of RT during the (neo)adjuvant treatment.

For O2, initial treatment was defined as the first treatment initiated within 180 days (6 months) after the date of diagnosis and any associated treatment initiated within a defined period as described in **Supplementary Table 2**. For CAS, initial treatment was defined as the first treatment initiated within a period of 30 days before until 180 days (6 months) after the date of diagnosis and any associated treatment initiated within a defined period as described in **Supplementary Table 2**; this approach was required to accommodate patients with a start of treatment date before their date of diagnosis since date of diagnosis was reliant on pathological assessment. For VONKOdb, initial treatment was defined as the first treatment initiated within the period of 15 days before until 180 days (6 months) after the date of diagnosis and any associated treatment initiated within a defined period as described in **Supplementary Table 2**; this approach was required to accommodate patients with no recorded day of diagnosis, for whom a proxy date of diagnosis was automatically set to the 15th of the month.

# Supplementary Table 2 Initial treatment categories for patients with incident stage IA-IIIC NSCLC

| Hierarchy order | Initial treatment category | Definition |
| --- | --- | --- |
| 1 | Surgery alone | First treatment recorded is surgery, AND patient has no record of SACT up to 84 days after surgery date, AND patient has no record of RT up to 84 days after surgery date. |
| 2 | Surgery +  adjuvant SACT | First treatment recorded is surgery, AND patient has record of *(adjuvant)* SACT up to 84 days after surgery date, AND patient has no record of RT up to 180 days after SACT start date. |
| 3 | Surgery +  adjuvant RT | First treatment recorded is surgery, AND patient has record of RT start up to 84 days after surgery date, AND patient has no record of SACT up to 180 days after RT start date. |
| 4 | Surgery +  adjuvant  SACT + RT | Either of the following applies:  (1) First treatment recorded is surgery, AND patient has a record of RT starting in the 84-day period after surgery date, AND patient has a record of SACT starting in the 180-day period after RT start date; OR  (2) First treatment recorded is surgery, AND patient has a record of SACT starting in the 84-day period after surgery date, AND patient has a record of RT starting in the 180-day period after SACT start date. |
| 5 | Surgery + neoadjuvant SACT | First treatment recorded is *(neoadjuvant)* SACT, AND patient has record of surgery up to 150 days after 1st SACT regimen start date, AND patient has no record of RT before surgery date. |
| 6 | Surgery +  perioperative SACT (± RT) | First treatment recorded is SACT *(before surgery)*, AND patient has record of surgery up to 150 days after 1st SACT regimen start date, AND patient has either a record of new SACT administration up to 84 days after the date of surgery or the end of the first SACT recorded after the surgery date.  *Note: This category allows a record of RT to occur during the (neo)adjuvant treatment.* |
| 7 | Surgery + neoadjuvant RT | First treatment recorded is RT, AND patient has record of surgery up to 90 days after 1st *(neoadjuvant)* RT regimen start date, AND patient has no record of SACT before surgery. |
| 8 | Surgery + neoadjuvant  SACT + RT | Either of the following applies:  (1) First treatment recorded is (*neoadjuvant*) SACT AND patient has record of RT start date up to 42 days after SACT start date, AND patient has record of surgery date up to 90 days after RT start date; OR  (2) First treatment is RT, AND patient has *(neoadjuvant*) SACT start date up to 42 days after RT start date, AND patient has record of surgery up to 90 days after SACT start date. |
| 9 | RT alone | First treatment recorded is RT, AND patient has no record of surgery up to 90 days after RT start date, AND patient has no record of SACT up to 90 days after RT start date. |
| 10 | SACT + palliative RT | Either of the following applies:  (1) First treatment recorded is RT with palliative intent, AND patient has record of SACT starting in the 90-day period after 1st RT start date AND patient has no surgery up to 180 days after 1st SACT start date; OR  (2) First treatment recorded is SACT, AND patient has record of RT with palliative intent starting in the 150-day period after 1st SACT regimen start date AND patient has no surgery up to 180 days after 1st SACT start date. |
| 11 | Sequential CRT | First treatment recorded is SACT, AND patient has a record of RT with curative/radical intent AND RT start date is after SACT end date, AND the RT start date is recorded in the 42-day period after SACT end date, AND patient has no record of surgery in the 90-day period after the end date of RT. |
| 12 | Concurrent CRT | First treatment recorded is SACT, AND patient has a record of RT with curative/radical intent AND RT start date is before SACT end date, AND patient has no record of IO starting in the 42-day period after the end date of RT, AND patient has no record of surgery in the 90-day period after the end date of RT. |
| 13 | Other curative SACT + RT | *Any curative SACT and RT treatment identified that was not classified as chemoradiation by the algorithm as defined above in points 11 & 12:*  Either of the following applies:  (1) First treatment recorded is RT with curative/radical intent, AND patient has a record of SACT starting in the 90-day period after 1st RT start date, AND patient has no record of surgery in the 180-day period after 1st SACT start date; OR  (2) First treatment recorded is SACT, AND patient has a record of RT with curative/radical intent, AND RT start date is in the 150-day period after 1st SACT start date, AND patient has no record of surgery in the 180-day period after 1st SACT start date. |
| 14 | Concurrent  CRT + IO | First treatment record is SACT, AND patient has record of RT with curative/radical intent AND RT start date is before SACT end date, AND patient has record of IO start date up to 42 days after the last RT administration, AND patient has no record of surgery up to 90 days after the end of RT. |
| 15 | SACT + RT (unknown intent) | Either of the following applies:  (1) First treatment recorded is RT, AND patient has a record of SACT starting in the 90-day period after 1st RT start date, AND patient has no record of surgery in the 180-day period after 1st SACT start date; OR  (2) First treatment recorded is SACT, AND patient has record of RT starting in the 150-day period after 1st SACT start date, AND patient has no record of surgery in the 180-day period after 1st SACT start date. |
| 16 | SACT alone | First treatment record is SACT, AND patient has no record of RT up to 150 days after 1st SACT start date, AND patient has no record of surgery up to 150 days after SACT start date. |
| 17 | Untreated | *No treatment received within first 6 months after diagnosis:*  Patient has no record of surgery up to 180 days after diagnosis date, AND patient has no record of SACT up to 180 days after diagnosis date, AND patient has no record of RT up to 180 days after diagnosis date. |
| 18 | Unclassified | Patient receives treatment that does not fall into any of the above categories. |

CRT, chemoradiotherapy; IO, immunotherapy; NSCLC, non-small cell lung cancer; RT, radiotherapy; SACT, systemic anticancer therapy.

# Supplementary Table 3 Tumor and node characteristics at diagnosis for patients with stage III NSCLC^a^

|  | **O2 (Canada)** | **CAS (England)** | **VONKOdb (Germany)** |
| --- | --- | --- | --- |
| **Stage IIIA** | **n = 1656** | **n = 14,295** | **n = 5083** |
| Tumor stage, n (%)  T0  T1  T1a  T1b  T1c^b^  T2  T2a  T2b  T3  T4  Tx  Missing | 0  0  91 (5.5)  109 (6.6)  26 (1.6)  24 (1.5)  365 (22.0)  193 (11.7)  447 (27.0)  401 (24.2)  0  0 | PM  47 (<1)  485 (3.4)  784 (5.5)  –  353 (2.5)  2365 (16.5)  1134 (7.9)  4502 (31.5)  4264 (29.8)  0  SM | 0  69 (1.4)  117 (2.3)  268 (5.3)  232 (4.6)  237 (4.7)  669 (13.2)  360 (7.1)  1140 (22.4)  1958 (38.5)  33 (<1)  0 |
| Node stage, n (%)  N0-N1  N2  N3  Nx  Missing | 585 (35.3)  1071 (64.7)  0  0  0 | 6217 (43.5)  7996 (55.9)  10 (<1)  0  72 (<1) | 2732 (53.7)  2321 (45.7)  0  30 (<1)  0 |
| **Stage IIIB** | **n = 958** | **n = 10,026** | **n = 3780** |
| Tumor stage, n (%)  T0  T1  T1a  T1b  T1c^b^  T2  T2a  T2b  T3  T4  Tx  Missing | PM  0  20 (2.1)  39 (4.1)  15 (1.6)  PM  115 (12.0)  49 (5.1)  186 (19.4)  506 (52.8)  19 (2.0)  0 | SM  46 (<1)  215 (2.1)  331 (3.3)  –  123 (1.2)  823 (8.2)  385 (3.8)  1813 (18.1)  5996 (59.8)  PM  284 (2.8) | 0  37 (<1)  37 (<1)  100 (2.7)  77 (2.0)  109 (2.9)  182 (4.8)  134 (3.5)  990 (26.2)  2054 (54.3)  60 (1.6)  0 |
| Node stage, n (%)  N0-N1  N2  N3  Nx  Missing | PM  498 (52.0)  459 (47.9)  0  0 | 15 (<1)  5515 (55.0)  4456 (44.4)  0  40 (<1) | 2 (<1)  2651 (70.1)  1093 (28.9)  34 (<1)  0 |
| **Stage IIIC** | **n = 86** | **n = 1167** | **n = 1073** |
| Tumor stage, n (%)  T0  T1  T1a  T1b  T1c^b^  T2  T2a  T2b  T3  T4  Tx  Missing | 0  0  0  0  0  0  0  0  38 (44.2)  48 (55.8)  0  0 | 0  0  0  0  –  0  0  0  SM  812 (69.6)  0  PM | 0  0  0  0  0  0  0  0  334 (31.1)  729 (67.9)  10 (<1)  0 |
| Node stage, n (%)  N0-N1  N2  N3  Nx  Missing | 0  0  86 (100.0)  0  0 | 0  PM  SM  0  0 | 0  0  1064 (99.2)  9 (<1)  0 |

CAS, Cancer Analysis System; NSCLC, non-small cell lung cancer; O2, Oncology Outcomes; PM, primary masked data; SM, secondary masked data; VONKOdb, Oncological Health Care Research Database.

Per specific data source requirements, individual categories including between 1 and 9 patients at O2, between 1 and 5 patients at CAS, and between 1 and 4 patients at VONKOdb underwent primary data masking. Additional secondary data masking was performed where necessary to prevent unmasking of categories undergoing primary data masking.
^a^ TNM stage was assigned at the date of NSCLC diagnosis. There were no restrictions on the source data used for TNM staging (clinical vs. post-surgical/pathological staging). For O2, TNM stage was derived only from clinical staging for this analysis. For CAS and VONKOdb, TNM stage could have been derived from either clinical staging or post-surgical/pathological staging.

^b^ Data for tumor stage T1c was not collected at CAS.

# Supplement References

1. Office for National Statistics. Population estimates for the UK, England, Wales, Scotland, and Northern Ireland Statistical bulletins. <https://www.ons.gov.uk/peoplepopulationandcommunity/populationandmigration/populationestimates/bulletins/annualmidyearpopulationestimates/mid2023#:~:text=1.-,Main%20points,or%20Northern%20Ireland%20(0.5%25>). Accessed 27 Jan 2025.

2. Health Data Insight. CAS Explorer: data visualisation <https://healthdatainsight.org.uk/project/cas-explorer/>. Accessed 1 June 2023.

3. The National Cancer Registration and Analysis Service (NCRAS). CAS Explorer. <https://www.cancerdata.nhs.uk/explorer>. Accessed 1 June 2023.

4. Katalinic A, Halber M, Meyer M, Pfluger M, Eberle A, Nennecke A, Kim-Wanner SZ, Hartz T, Weitmann K, Stang A, et al. Population-based clinical cancer registration in Germany. Cancers (Basel). 2023;15(15):3934.
